# ^1^IFN-α Modulates Memory Tfh Cells and Memory B Cells in Mice, Following Recombinant FMDV Adenoviral Challenge

**DOI:** 10.3389/fimmu.2020.00701

**Published:** 2020-04-29

**Authors:** Xiangguo Duan, Peng Sun, Yaru Lan, Chunxiu Shen, Xiaoyu Zhang, Shaozhang Hou, Jian Chen, Bin Ma, Yuhan Xia, Chunxia Su

**Affiliations:** ^1^School of Basic Medical Sciences, Ningxia Medical University, Yinchuan, China; ^2^Department of Laboratory Medicine, College of Clinical Medicine, Ningxia Medical University, Yinchuan, China; ^3^Department of Laboratory Surgery, General Hospital of Ningxia Medical University, Yinchuan, China; ^4^Guolong Hospital, Yinchuan, China; ^5^Department of Oncology Surgery, The First People’s Hospital of Yinchuan, Yinchuan, China; ^6^General Hospital of Ningxia Medical University, Yinchuan, China

**Keywords:** IFN-α, foot-and-mouth disease recombinant adenovirus vaccine, immune memory, memory Tfh cell, memory B cell

## Abstract

Follicular helper T (Tfh) cells regulate high-affinity antibody production. Some findings have indicated that Tfh cells could be differentiated into memory cells. Here we have investigated the effects of IFN-α, as an adjuvant, on the generation of memory Tfh cell and memory B cell responses. The data showed that adenoviral vectors expressing: (i) foot-and-mouth disease virus (FMDV) VP1 proteins and porcine IFN-α, or (ii) porcine IFN-α alone, potently enhanced the generation of memory Tfh cells, especially the CCR7^*lo*^ memory Tfh subset. Upon rechallenge with FMD recombinant adenoviral vaccines, IFN-α enhances Tfh cells activity, rapidly upregulating their signature Bcl-6, CXCR5, and IL-21 markers. The results suggest that IFN-α enhances the levels of the transcription factor Bcl-6 within Tfh cells, potentially by regulating STAT1. Additionally, IFN-α substantially increased the number of IgG1^+^ and CD86^+^ memory B cells, which are responsible for inducing the rapid effector functions of memory Tfh cells after vaccine reactivation, establishing the close relationship between memory B cell and memory Tfh cell subsets. In brief, IFN-α enhances the potency of FMD recombinant adenoviral vaccines to induce memory Tfh and memory B cell responses, thus elevating serum antibody titers. IFN-α administration therefore represents an attractive strategy for enhancing responses to vaccination.

## Introduction

The most effective vaccines in use today rely on the long-term protection elicited by high-affinity memory B cells and long-lived plasma cells, which are associated with germinal centers (GC) (1). Follicular helper T (Tfh) cells, a newly discovered CD4^+^ T cell subset, can regulate humoral immune responses in the body and drive cells to migrate into peripheral lymphoid follicles. Tfh cells also help B cell proliferation and differentiation, the generation of GCs (2–4), and stimulate B cells to produce antibodies and promote antibody class switching (5). Therefore, Tfh cells are critical in GC formation and the production of high-affinity antibodies (6). B cell lymphoma 6 (Bcl-6), a Tfh cell-specific transcription factor, is a key regulator of Tfh cell differentiation and function (7). Bcl-6 induces the expression of the chemokine receptor CXCR5, a hallmark of Tfh cells, which promotes their migration into CXCL13 rich areas such as B cell follicles (8). The cytokine IL-21, secreted by Tfh cells, is a major factor in Tfh cell effector function. IL-21 functions by binding to the IL-21 receptor on the surface of B cells in the GC (9). Meanwhile, Tfh cells themselves express IL-21 receptors, thus affecting the differentiation and migration of Tfh cells via autocrine signaling (10, 11). Because of their central role in the production of long-lasting humoral immunity, Tfh cells represent an interesting target for rational vaccine design. Novel or modified adjuvants may prove to be an effective strategy for skewing helper T cell differentiation toward the Tfh cell subset and promoting GC responses. Type I interferons (IFN-I) represent a family of cytokines with antiviral and immunomodulatory activities (12). Researchers have previously identified IFN-I as a natural adjuvant that selectively supports the generation of lymph node resident Tfh cells (13). Our previous work revealed the potent adjuvant activity of IFN-α, which enhanced the generation of Tfh cells and regulated humoral immunity by promoting GC reactions and antibody responses (14).

Some studies have revealed that Tfh cells are capable of differentiating into memory cells. Memory Tfh cells strongly downregulated their signature Bcl- 6-, CXCR5-, and PD-1-encoding genes in the memory phase (2, 15). Upon rechallenge with antigen, they once more rapidly upregulated these markers. Memory Tfh cells retain their capacity to recall their Tfh-specific effector functions upon antigen reactivation to provide help for B cell responses, and therefore play an important role in prime and boost vaccination or during recall responses to infection (16). These findings have important implications for rational vaccine design, where improving the generation and engagement of memory Tfh cells could be used to enhance vaccine-induced protective immunity. Thus, it is critical to design rational prime and boost strategies for the optimal generation of Tfh memory cells.

Further research (in both humans and animal models) into precisely how we can manipulate memory Tfh cells to improve vaccine responses, may enable us to address some of the challenges currently facing the development of effective vaccines. The present study shows that IFN-α can promote the development of immune memory, including memory Tfh cells and memory B cells, in response to the foot-and-mouth disease vaccine, and thus promote rapid immune reactivation on repeated antigen exposure.

## Materials and Methods

### Mice and Vaccination Regimens

A total of 6 to 8 weeks old female BALB/c mice (from Shanghai Xipuer-Beikai Experimental Animal Co., Ltd) were used in all immunization experiments. All mice were randomly divided into six groups (20 mice per group, 5 mice at each time point within group), respectively injected with 5 × 10^8^ TU/ml recombinant adenovirus rAd (reconstituted Adenovirus vector type 5), rAd5VP1 (VP1 is a one of envelop protein from Foot and Mouth Virus), rAd5poIFN-α (IFN-α reconstituted Adenovirus vector type 5), rAd5VP1 + rAd5poIFN-α (combined rAd5VP1 and rAd5poIFN-α), rAd5VP1-2A- poIFN-α (co-expression VP1 and poIFN-α reconstituted Adenovirus vector type 5), and FMDV inactivated vaccine (commercial vaccine). The animals received a booster infection 15 days after the initial immunization. Mice were harvested 30, 60, and 90 days after priming. In addition, the mice were immunized again the same way 90 days later, and the mice were harvested 3 days later.

### Flow Cytometry Analysis

Single cell suspensions of peripheral blood mononuclear cells (PBMCs) derived from the mouse spleen were stained for 30 min at 4°C with anti-mouse CD4 (RM4-5), anti-mouse CXCR5 (2G8), anti-mouse CD44 (IM7), anti-mouse CD62L (MEL-14), anti-mouse CXCR3 (CXCR3-173), anti-mouse CCR6 (140706), anti-mouse CD38 (90/CD38), anti-mouse B220 (RA3-6B2), anti-mouse GL-7 (GL7), anti-mouse IgG1 (A85-1), anti-mouse CD86 (GL1), anti-mouse Bcl-6 (K112-91), anti-mouse IL-21 (mhalx21), anti-mouse IFN-γ (XMG1.2), and anti-mouse IL-4 (11B11). All of the listed antibodies, with the exception of anti-mouse IL-21 (which was sourced from eBioscience), were purchased from BD Pharmingen. For the detection of intracellular factors by flow cytometry, PBMCs were stimulated with a combination of phorbol myristate acetate (PMA), ionomycin calcium salt, and monensin, prior to performing intracellular staining. Cell acquisition was performed on a FACSCelesta^TM^ Flow Cytometer (BD Biosciences).

### Quantitative Real-Time PCR

PBMC RNA was isolated with an RNAsimple Total RNA Kit according to the manufacturer’s guidelines (TIANGEN) and reverse transcribed into cDNA using the First Strand cDNA Synthesis Kit (Thermo Fisher Scientific). For the mRNA expression assays, the following probes were used (all from Applied Thermo Fisher Scientific): BCL-6, Mm00477633_m1; STAT1, Mm01257286_m1; and β-actin, Mm02619580_m1. The reactions were set up following manufacturer’s guidelines and the mRNA expression was evaluated by real-time reverse transcription polymerase chain reaction (RT-PCR) analysis.

### Enzyme-Linked Immunosorbent Assay (ELISA)

Mice serum was harvested after immunization. Total IgG, IgG1, IgG2a, and IgG2b were assayed using the mouse ELISA Kit (eBioscience), according to the manufacturer’s instructions. Absorbance at 450 nm was measured using a spectrometer enzyme-labeled instrument (Bio-Rad).

### Statistical Analysis

Flow cytometry results were analyzed using Flowjo software (Tree Star, Inc.). All statistical tests were performed using Prism 5 (GraphPad Software, Inc.). The results were expressed as means ± SD of the indicated number of experiments. *P* values were calculated using *t* tests or two-way ANOVA, with a 95% confidence interval. *P* values below 0.05 were considered statistically significant (^∗^*P* < 0.05, ^∗∗^*P* < 0.01, ^∗∗∗^*P* < 0.001, and ^****^P < 0.0001).

## Results

### IFN-α Enhances the Generation of Memory Tfh Cells, Induced by Recombinant Adenoviruses

**FIGURE 1:**
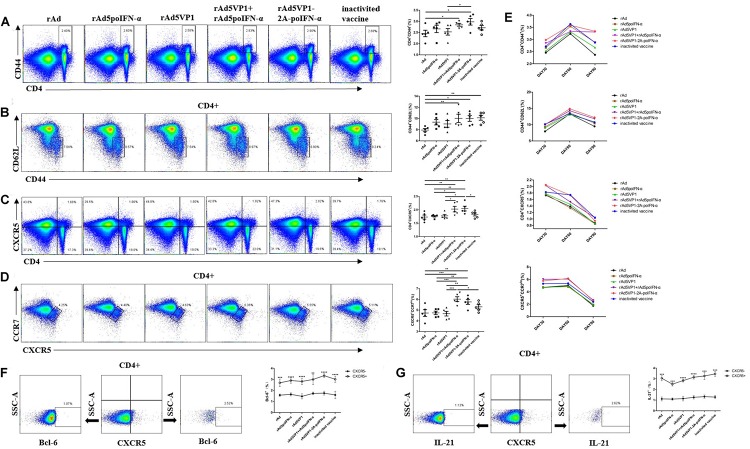
IFN-a enhances the generation of memory Tfh cells. **(A–D)** Thirty days after the immunization, representative dot plot from flow cytometric analysis of CD44^+^CD4^+^ T cells, CD62L^-^CD44^+^ T cells (gating within CD4 + T cells), CXCR5^+^CD4^+^ T cells, and CCR7^*lo*^ CXCR5^+^ T cells (gating within CD4 + T cells). The percentage of four subsets of CD4^+^T cells. One way analysis of variance (ANOVA). **(E)** The percentage of four subsets of CD4 + T cells after 30, 60, and 90 days of immunization. **(F,G)** Gating strategy for the analysis of CXCR5^+^CD4^+^ T cell subsets and the percentage of Bcl-6^+^ and IL-21^+^ cells within CXCR5^+^ and CXCR5^-^CD4^+^ T cell compartments after 90 days of immunization. Paired *t*-test. *P < 0.05, **P < 0.01, ***P < 0.001, and ****P < 0.0001.

Our previous work showed that porcine IFN-α potently enhanced the generation of Tfh cells induced by FMD recombinant adenovirus vaccines, and thus increased the expression of Bcl-6 mRNA and the secretion of IL-21 in the serum (14). It was revealed that Tfh cells are able to survive as memory cells, with the vast majority residing in the spleen and peripheral lymph nodes (17). To address whether IFN-α up-regulates the generation of memory Tfh cells, BALB/c mice were immunized with either adenoviral vectors expressing FMDV VP1 alone (rAd5VP1) or co-expressing VP1 and IFN-a (rAd5VP1-2A-poIFN-α). In addition, BALB/c mice were immunized simultaneously with adenoviral vectors expressing FMDV VP1 and those expressing porcine IFN-α. The splenocytes were harvested on days 30, 60, and 90 after immunization, and the activated CD4^+^ T cells, memory CD4^+^ T cells, and memory Tfh cells (CXCR5^+^CD4^+^) were enumerated and characterized by multiple-color flow cytometry. As shown in Figures 1A–C, we found an marked increase in the frequency of activated CD4^+^ T cells, memory CD4^+^ T cells, and memory Tfh cells in mice immunized with recombinant adenoviruses, which was sustained for at least 90 days post immunization (Figure 1E). The CCR7^*lo*^ Tfh cell subset provides a biomarker for monitoring protective antibody responses during infection or vaccination. Therefore, we subsequently quantified CCR7^*lo*^CXCR5^+^CD4^+^ T cells on days 30, 60, and 90 after immunization. We found that IFN-α enhanced the recombinant adenovirus-induced generation of CCR7^*lo*^CXCR5^+^CD4^+^ T cells (Figure 1D), which was sustained for at least 90 days following immunization (Figure 1E). The result confirmed that IFN-α enhances the generation of memory Tfh cells induced by recombinant adenoviruses. Bcl-6, a master regulator of Tfh cell generation, is required for Tfh memory cell maintenance and the subsequent establishment of humoral memory. To this end, we also monitored the intracellular expression of Bcl-6 in Tfh cells at day 90 after immunization, and found that splenic memory CXCR5^+^ Th cells expressed low levels of Bcl-6. However, Bcl-6 expression levels were significantly higher in these memory CXCR5^+^ Th cells than in their CXCR5^–^ Th cell counterparts. Similarly, we observed that IFN-α enhanced the expression levels of Bcl-6 in memory Tfh cells (Figure 1F). It is therefore likely that Tfh memory cells, endowed with an enhanced potential to produce IL-21, paired with higher CXCR5 and lower CCR7 expression (Figure 1G), should enable this unique subset of Th cells to provide more efficient help for antigen-specific B cells than their non-Tfh counterparts.

### IFN-α Enhances Chemokine Receptor Expression by Memory Tfh Cells Following Recombinant Adenoviral Exposure

**FIGURE 2:**
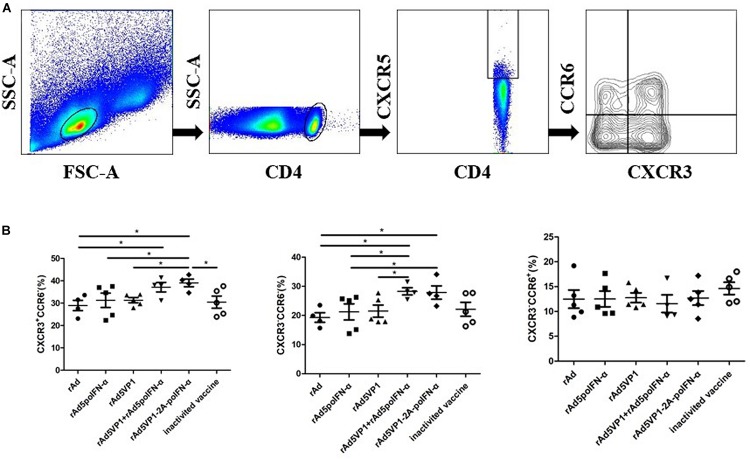
IFN-α enhances chemokine receptor expression by memory Tfh cells. **(A)** Gating strategy for the analysis of CXCR3 and CCR6 among of CXCR5^+^CD4^+^ T cells. **(B)** The percentage of CXCR3^+^CCR6^-^, CXCR3^-^CCR6^-^, and CXCR3^-^CCR6^+^ T cells within CXCR5^+^CD4^+^ T cell compartments after 90 days of immunization. One way analysis of variance (ANOVA). *P < 0.05.

We next assessed the expression of other chemokine receptors at the surface of memory Tfh cells. We monitored the expression of the chemokine receptors CXCR3 and CCR6, whose differential expression defines the following Tfh cell subsets: cTfh1 (CXCR3^+^CCR6^–^), cTfh2 (CXCR3^–^CCR6^–^), and cTfh17 (CXCR3^–^CCR6^+^) (Figure 2A). We found that the proportions of cTfh1 and cTfh2, but not cTfh17, cells were significantly increased in mice immunized with recombinant adenoviruses (Figure 2B). The results reveal that IFN-α enhances the generation of cTfh1 and cTfh2 memory Tfh cell subtypes following recombinant adenoviral exposure.

### IFN-α Enhances Memory B Cell Generation Following Recombinant Adenoviral Exposure

**FIGURE 3:**
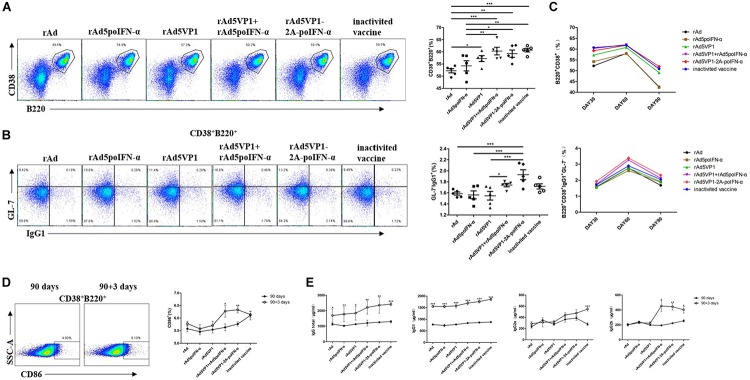
IFN-α enhances memory B cell generation. **(A,B)** Thirty days after the immunization, representative dot plot from flow cytometric analysis of B220^+^CD38^+^ B cells and IgG1^+^GL-7^-^ B cells. The percentage of two cells. One way analysis of variance (ANOVA). **(C)** The percentage of two subsets of B cells after 30, 60, and 90 days of immunization. **(D)** Representative dot plot from flow cytometric analysis of CD86^+^ B cells. The percentage of CD86^+^ B cells among of B220^+^CD38^+^ B cells before and after rechallenge. Paired *t*-test. **(E)** Serum levels of total IgG, IgG1, IgG2a, and IgG2b were analyzed by ELISA. Paired *t*-test. *P < 0.05, **P < 0.01, and ***P < 0.001.

To test whether memory B cell numbers correlated with those of memory Tfh cells, we analyzed memory B cells on days 30, 60, and 90 after immunization. Consistently, the proportion of memory B cells and IgG1 levels were significantly increased in mice immunized with recombinant adenoviruses, which was sustained for at least 90 days following immunization (Figures 3A–C). The result reveal that IFN-α enhances the generation of memory B cells induced by recombinant adenoviruses. CD86^+^ memory B cells can become antibody-secreting effector plasma cells after rechallenge with antigen. We therefore also checked CD86 expression on memory B cells in addition to their antibody production capacity, and found that they rapidly upregulated both functions after rechallenge with antigen (Figures 3D,E).

### IFN-α Enhances the Memory Tfh Cell Numbers Contributing to Recall Responses Following Subsequent Antigen Challenge

**FIGURE 4:**
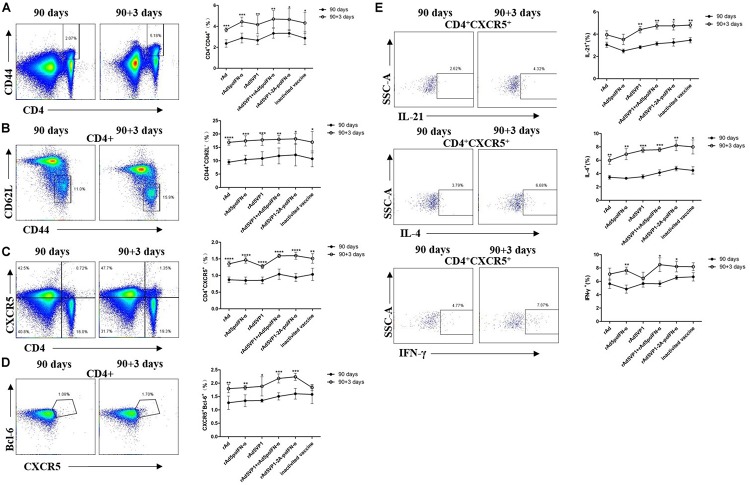
IFN-α enhances the memory Tfh cell numbers contributing to recall responses following subsequent antigen challenge. **(A–D)** Representative dot plot from flow cytometric analysis of CD44^+^CD4^+^ T cells, CD62L^-^CD44^+^ T cells (gating within CD4 + T cells), CXCR5^+^CD4^+^ T cells, and CXCR5^+^Bcl-6^+^ T cells (gating within CD4 + T cells). The percentage of four subsets of CD4 + T cells before and after rechallenge. **(E)** Representative dot plot from flow cytometric analysis of IL-21^+^, IL-4^+^ and IFN-γ^+^ T cells. The percentage of IL-21^+^, IL-4^+^, and IFN-γ^+^ T cells among of CXCR5^+^CD4^+^ T cells before and after rechallenge. Paired *t*-test. *P < 0.05, **P < 0.01, ***P < 0.001, and ****P < 0.0001.

To investigate whether long-lived memory Tfh cells can contribute to recall responses following subsequent antigen challenge, we analyzed CD4^+^ T cells and Tfh memory cells 3 days after their reactivation *in vivo*. We found that IFN-α enhanced memory Tfh cell activation after rechallenge with antigen. Upon rechallenge with FMD recombinant adenoviral vaccines, memory Tfh cells once again rapidly upregulated their signature Bcl-6 and CXCR5 markers, which were both strongly downregulated in the resting memory phase (Figures 4A–D). Thus, these data demonstrate that Bcl-6 is induced at a much earlier timepoint in the memory Tfh cell response than in naïve T cells, and that this rapid Bcl-6 upregulation correlates well with IL-21 expression (Figure 4E). Besides IL-21, we also measured the intracellular production of IL-4 and IFN-γ by memory Tfh cells 3 days after antigenic restimulation, and found that these were also rapidly upregulated (Figure 4E).

### IFN-α Enhances Memory Tfh Cell Formation, Potentially by Regulating the Transcription Factors Bcl-6 and STAT1

**FIGURE 5:**
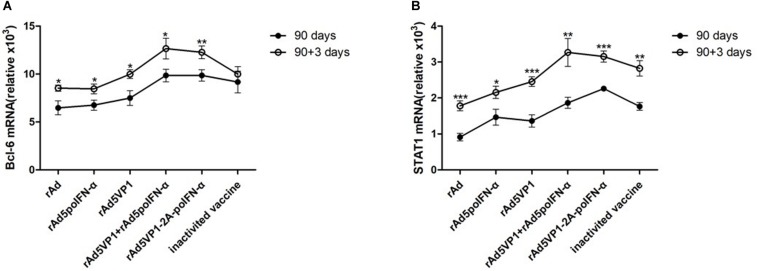
IFN-α enhances memory Tfh cell formation, potentially by regulating the transcription factors Bcl-6 and STAT1. **(A,B)** Bcl-6 and STAT1 mRNA was measured and normalized simultaneously with mouse β-actin endogenous controls by quantitative Real-Time PCR before and after rechallenge. Paired *t*-test. *P < 0.05, **P < 0.01, and ***P < 0.001.

As Bcl-6 is a master regulator of Tfh cell generation, we checked Bcl-6 mRNA levels, and found that they consistently upregulated after rechallenge with antigen (Figure 5A). STAT1 acts as an important Tfh cell transcription factor, and directly regulates the expression of Bcl-6. We also observed STAT1 upregulation after rechallenge with antigen (Figure 5B). These data indicate that IFN-α enhances memory Tfh cell differentiation potentially by regulating the transcription factors Bcl-6 and STAT1.

## Discussion

The success of most vaccines relies on the generation of antibodies to provide protection against subsequent infection. Antibody affinity maturation and the generation of B cell memory occur within the specialized GC microenvironment within the B cell follicles of secondary lymphoid organs (18). Tfh cells provide growth and differentiation signals to GC B cells and mediate the positive selection of high-affinity B cell clones in the GC, thereby determining which B cells exit the GC as plasma cells and memory B cells (19–21). Because of their central role in the production of long-lasting humoral immunity, Tfh cells represent an interesting target for rational vaccine design. Strategies aiming to augment Tfh cell numbers may therefore represent a rational approach to enhancing vaccine responses. Overall, subcutaneous immunization promotes the development of memory Tfh cells (15, 17). Our previous study has shown that the numbers of Tfh cells and GC B cells positively correlate, as do the numbers of memory Tfh cells after FMDV recombinant adenoviral vaccination in the present study. We have also previously demonstrated that IFN-α could enhance the numbers of Tfh cells, GC B cells, and memory Tfh cells.

Upon rechallenge with FMD recombinant adenoviral vaccines, Tfh cells rapidly upregulate their signature Bcl- 6 and CXCR5 markers, which are otherwise strongly downregulated in the resting memory phase (7, 8). Here we have shown that IFN-α could enhance the expression of these molecules and promote memory Tfh activation after rechallenge with antigen. Taken together, these data demonstrate that Bcl-6 is induced at a much earlier timepoint in memory Tfh cell activation than in naïve T cells, and that this rapid Bcl-6 upregulation correlates well with IL-21 expression. Here, we were also surprised to discover that IFN-α enhances the differentiation of cTfh1 and cTfh2, but not cTfh17 cell subsets. Some studies have implied that the CCR7^*lo*^ Tfh cell subset provides a biomarker for monitoring protective antibody responses during infection or vaccination, and pathogenic antibody responses in autoimmune disease (22). We have shown that FMDV recombinant adenoviruses could induce generation of CCR7^*lo*^CXCR5^+^CD4^+^ T cells, while IFN-α enhanced the numbers of these cells. These data indicate that IFN-α administration could represent an effective strategy for the generation of CCR7^*lo*^ memory Tfh cells to enhance vaccine responses.

Many cytokines that drive helper T cell specification bind type I/II cytokine receptors, which activate the JAK/STAT signaling pathway, culminating in effector cell differentiation (23, 24). Previous studies have shown the direct effects of type I IFNs on Tfh differentiation, by inducing Bcl-6 upregulation and promoting CXCR5 expression (14). The ability of IFN-α/β to drive these Tfh cell features was entirely STAT1-dependent, which binds directly at the Bcl-6 locus (25). The results of the present study also confirm the above findings.

Another study has shown that in these recall responses, antigen-specific memory B cells greatly contribute to the rapid induction of Bcl-6 and IL-21 in memory Tfh cells (26). Here, we have demonstrated that IFN-α also enhances the generation of memory B cells, IgG1 memory B cells, and the production of antibodies induced by recombinant adenoviruses. Studie has shown that IgG1 memory B cells are more likely to differentiate into plasma cells than IgM memory B cells or naive cells (27). Furthermore, their proliferation and differentiation into plasma cells may require direct T cell/B cell interactions. Some reports have found that CD4^+^ T cells are close to contracted GC on the 60th day and are present in the vicinity of IgG1 memory B cells. It is speculated that long-lived memory Tfh cells that make up a portion of these CD4^+^ T cells, are mainly responsible for activating IgG1 memory B cells (28). After antigen re-exposure, memory B cells develop into short-lived effectors capable of rapid antibody production. In addition, memory B cells reenter GCs, resulting in the generation of affinity-matured long-lived plasma cells (LLPCs) and memory B cells. This process depends on costimulation and cytokine production by Tfh cells. As a general rule in mice, IFN-γ^+^ Tfh cells can be found in conjunction with IgG2a^+^ B cells, whereas IL-4^+^ Tfh cells were more likely to be paired with IgG1^+^ B cells (29–31). Here, we have shown that IFN-α enhances both IFN-γ^+^ Tfh cells and IL-4^+^ Tfh cells activity. IFN-α also enhances CD86 expression in memory B cells before they become antibody-secreting effector plasma cells. CD86 is then rapidly upregulated on these cells after rechallenge with antigen. Increased antigen presentation by B cells also increases the capacity of Tfh cells to produce the cytokines IL-4 and IL-21. An enhanced potential to produce IL-21, paired with higher CXCR5 and lower CCR7 expression levels, should therefore enable memory Tfh cells to provide more efficient help for antigen-specific B cells than their non-Tfh counterparts (32).

Our results demonstrate that memory Tfh cells can survive for long periods and are rapidly reactivated, upon FMDV recombinant adenoviral recall, to differentiate into effector Tfh cells. It has been shown that this step is dependent on memory B cells, suggesting that memory B cells may directly activate memory Tfh cells in the follicle (32). It is generally accepted that CXCR5^+^ memory Tfh cells preferentially home to the T-B border and the follicle to provide help to B cells (33). In agreement with these findings, we found that IFN-α could also enhance memory B cell-mediated immunity. Based on these data, we propose a strategy whereby IFN-α could enhance the anti-FMD recombinant adenoviral immune response by promoting cognate interactions between memory Tfh and memory B cells, with important implications for the development of better vaccines.

## Data Availability Statement

All datasets generated for this study are included in the article/supplementary material.

## Ethics Statement

All mouse procedures were approved by the Institutional Animal Care and Use Committee of the Ningxia Medical University (Ningxia, China, 2013-024).

## Author Contributions

CS had full access to all of the data in the study and took responsibility for the integrity of the data and the accuracy of the data analysis. XD and CS conceived and designed the study. All authors acquired the data, analyzed the data, interpreted the data, reviewed the manuscript, provided comments or suggestions, and read and approved the final manuscript.

## Conflict of Interest

The authors declare that the research was conducted in the absence of any commercial or financial relationships that could be construed as a potential conflict of interest.

## References

[1] Frasca D Diaz A Romero M Blomberg BB. The generation of memory B cells is maintained, but the antibody response is not, in the elderly after repeated influenza immunizations. *Vaccine.* (2016) 34:2834–40. 10.1016/j.vaccine.2016.04.023 27108193 PMC4876633

[2] Shulman Z Gitlin AD Targ S Jankovic M Pasqual G Nussenzweig MC T follicular helper cell dynamics in germinal centers. *Science.* (2013) 341:673–7. 10.1126/science.1241680 23887872 PMC3941467

[3] Crotty S. T follicular helper cell differentiation, function, and roles in disease. *Immunity.* (2014) 41:529–42. 10.1016/j.immuni.2014.10.004 25367570 PMC4223692

[4] Cannons JL Qi H Lu KT Dutta M Gomez-Rodriguez J Cheng J Optimal germinal center responses require a multistage T cell:B cell adhesion process involving integrins, SLAM-associated protein, and CD84. *Immunity.* (2010) 32:253–65. 10.1016/j.immuni.2010.01.010 20153220 PMC2830297

[5] McHeyzer-Williams LJ Pelletier N Mark L Fazilleau N McHeyzer-Williams MG. Follicular helper T cells as cognate regulators of B cell immunity. *Curr Opin Immunol.* (2009) 21:266–73. 10.1016/j.coi.2009.05.010 19502021 PMC2731669

[6] Zotos D Coquet JM Zhang Y Light A D’Costa K Kallies A IL-21 regulates germinal center B cell differentiation and proliferation through a B cell-intrinsic mechanism. *J Exp Med.* (2010) 207:365–78. 10.1084/jem.20091777 20142430 PMC2822601

[7] Rolf J Bell SE Kovesdi D Janas ML Soond DR Webb LM Phosphoinositide 3-kinase activity in T cells regulates the magnitude of the germinal center reaction. *J Immunol.* (2010) 185:4042–52. 10.4049/jimmunol.1001730 20826752

[8] Yu D Rao S Tsai LM Lee SK He Y Sutcliffe EL The transcriptional repressor Bcl-6 directs T follicular helper cell lineage commitment. *Immunity.* (2009) 31:457–68. 10.1016/j.immuni.2009.07.002 19631565

[9] Bessa J Kopf M Bachmann MF. Cutting edge: IL-21 and TLR signaling regulate germinal center responses in a B cell-intrinsic manner. *J Immunol.* (2010) 184:4615–9. 10.4049/jimmunol.0903949 20368279

[10] Linterman MA Beaton L Yu D Ramiscal RR Srivastava M Hogan JJ IL-21 acts directly on B cells to regulate Bcl-6 expression and germinal center responses. *J Exp Med.* (2010) 207:353–63. 10.1084/jem.20091738 20142429 PMC2822609

[11] Nurieva RI Chung Y Hwang D Yang XO Kang HS Ma L Generation of T follicular helper cells is mediated by interleukin-21 but independent of T helper 1, 2, or 17 cell lineages. *Immunity.* (2008) 29:138–49. 10.1016/j.immuni.2008.05.009 18599325 PMC2556461

[12] Theofilopoulos AN Baccala R Beutler B Kono DH. Type I interferons. (alpha/beta) in immunity and autoimmunity. *Annu Rev Immunol.* (2005) 23:307–36. 15771573 10.1146/annurev.immunol.23.021704.115843

[13] Cucak H Yrlid U Reizis B Kalinke U Johansson-Lindbom B. Type I interferon signaling in dendritic cells stimulates the development of lymph-node-resident T follicular helper cells. *Immunity.* (2009) 31:491–501. 10.1016/j.immuni.2009.07.005 19733096

[14] Su C Duan X Zheng J Liang L Wang F Guo L. IFN-alpha as an adjuvant for adenovirus-vectored FMDV subunit vaccine through improving the generation of T follicular helper cells. *PLoS One.* (2013) 8:e66134. 10.1371/journal.pone.0066134 23823532 PMC3688841

[15] Weber JP Fuhrmann F Hutloff A. T-follicular helper cells survive as long-term memory cells. *Eur J Immunol.* (2012) 42:1981–8. 10.1002/eji.201242540 22730020

[16] Hale JS Ahmed R. Memory T follicular helper CD4 T Cells. *Front Immunol.* (2015) 6:16. 10.3389/fimmu.2015.00016 25699040 PMC4313784

[17] Hale JS Youngblood B Latner DR Mohammed AU Ye L Akondy RS Distinct memory CD4+ T cells with commitment to T follicular helper- and T helper 1-cell lineages are generated after acute viral infection. *Immunity.* (2013) 38:805–17. 10.1016/j.immuni.2013.02.020 23583644 PMC3741679

[18] Ferretti E Ponzoni M Doglioni C Pistoia V. IL-17 superfamily cytokines modulate normal germinal center B cell migration. *J Leukoc Biol.* (2016) 100: 913–8. 27566830 10.1189/jlb.1VMR0216-096RR

[19] Kurosaki T Kometani K Ise W. Memory B cells. *Nat Rev Immunol.* (2015) 15:149–59. 10.1038/nri3802 25677494

[20] Vaccari M Franchini GT. Cell subsets in the germinal center: lessons from the macaque model. *Front Immunol.* (2018) 9:348. 10.3389/fimmu.2018.00348 29535724 PMC5834428

[21] De Silva NS Klein U. Dynamics of B cells in germinal centres. *Nat Rev Immunol.* (2015) 15:137–48. 10.1038/nri3804 25656706 PMC4399774

[22] He J Tsai LM Leong YA Hu X Ma CS Chevalier N Circulating precursor CCR7(lo)PD-1(hi) CXCR5(+) CD4(+) T cells indicate Tfh cell activity and promote antibody responses upon antigen reexposure. *Immunity.* (2013) 39:770–81. 10.1016/j.immuni.2013.09.007 24138884

[23] O’Shea JJ Plenge R. JAK and STAT signaling molecules in immunoregulation and immune-mediated disease. *Immunity.* (2012) 36:542–50. 10.1016/j.immuni.2012.03.014 22520847 PMC3499974

[24] O’Shea JJ Lahesmaa R Vahedi G Laurence A Kanno Y. Genomic views of STAT function in CD4+ T helper cell differentiation. *Nat Rev Immunol.* (2011) 11:239–50. 10.1038/nri2958 21436836 PMC3070307

[25] Nakayamada S Poholek AC Lu KT Takahashi H Kato M Iwata S Type I IFN induces binding of STAT1 to Bcl6: divergent roles of STAT family transcription factors in the T follicular helper cell genetic program. *J Immunol.* (2014) 192:2156–66. 10.4049/jimmunol.1300675 24489092 PMC3967131

[26] Ise W Inoue T McLachlan JB Kometani K Kubo M Okada T Memory B cells contribute to rapid Bcl6 expression by memory follicular helper T cells. *Proc Natl Acad Sci USA.* (2014) 111:11792–7. 10.1073/pnas.1404671111 25071203 PMC4136626

[27] Pape KA Taylor JJ Maul RW Gearhart PJ Jenkins MK. Different B cell populations mediate early and late memory during an endogenous immune response. *Science.* (2011) 331:1203–7. 10.1126/science.1201730 21310965 PMC3993090

[28] Aiba Y Kometani K Hamadate M Moriyama S Sakaue-Sawano A Tomura M Preferential localization of IgG memory B cells adjacent to contracted germinal centers. *Proc Natl Acad Sci USA.* (2010) 107:12192–7. 10.1073/pnas.1005443107 20547847 PMC2901464

[29] Reinhardt RL Liang HE Locksley RM. Cytokine-secreting follicular T cells shape the antibody repertoire. *Nat Immunol.* (2009) 10:385–93. 10.1038/ni.1715 19252490 PMC2714053

[30] Lu KT Kanno Y Cannons JL Handon R Bible P Elkahloun AG Functional and epigenetic studies reveal multistep differentiation and plasticity of in vitro-generated and in vivo-derived follicular T helper cells. *Immunity.* (2011) 35:622–32. 10.1016/j.immuni.2011.07.015 22018472 PMC3235706

[31] Fang D Cui K Mao K Hu G Li R Zheng M Transient T-bet expression functionally specifies a distinct T follicular helper subset. *J Exp Med.* (2018) 215:2705–14. 10.1084/jem.20180927 30232200 PMC6219743

[32] Bentebibel SE Lopez S Obermoser G Schmitt N Mueller C Harrod C Induction of ICOS+CXCR3+CXCR5+ TH cells correlates with antibody responses to influenza vaccination. *Sci Transl Med.* (2013) 5:176ra32. 10.1126/scitranslmed.3005191 23486778 PMC3621097

[33] MacLeod MK David A McKee AS Crawford F Kappler JW Marrack P. Memory CD4 T cells that express CXCR5 provide accelerated help to B cells. *J Immunol.* (2011) 186:2889–96. 10.4049/jimmunol.1002955 21270407 PMC3069687

